# Endoscopic management of gallstone large bowel obstruction at a sigmoid diverticular stricture secondary to cholecystoduodenal fistula

**DOI:** 10.1093/jscr/rjaf702

**Published:** 2026-03-16

**Authors:** Lawrence R Feng, Logan G Peter, Christopher D Barrett, Miguel A Matos, Reynold Henry

**Affiliations:** Department of Surgery, University of Nebraska Medical Center, S 42nd & Emile St, Omaha, NE 68198, United States; Department of Surgery, University of Nebraska Medical Center, S 42nd & Emile St, Omaha, NE 68198, United States; Department of Surgery, Division of Acute Care Surgery, University of Nebraska Medical Center, S 42nd & Emile St, Omaha, NE 68198, United States; Department of Surgery, Division of Acute Care Surgery, University of Nebraska Medical Center, S 42nd & Emile St, Omaha, NE 68198, United States; Department of Surgery, Division of Acute Care Surgery, University of Nebraska Medical Center, S 42nd & Emile St, Omaha, NE 68198, United States

**Keywords:** gallstone ileus, large bowel obstruction, sigmoid stricture, cholocystoduodenal fistula

## Abstract

Gallstone ileus is a rare cause of mechanical bowel obstruction, typically involving the small intestine. Large bowel obstruction (LBO) due to gallstone impaction is exceedingly uncommon, particularly at a sigmoid stricture. We present the case of a 76-year-old male with significant cardiac comorbidities who was managed non-operatively for acute cholecystitis complicated by cholecystoduodenal fistula. He subsequently re-presented with LBO from an impacted gallstone at a sigmoid diverticular stricture. Multidisciplinary endoscopic intervention using mechanical lithotripsy avoided surgical resection. This case highlights the potential for nonoperative management of gallstone LBO in select patients and emphasizes the utility of advanced endoscopic techniques in the setting of challenging anatomy. It also contributes to the limited but growing body of literature describing colonic gallstone ileus, particularly in the context of diverticular disease.

## Introduction

Gallstone ileus is an uncommon cause of bowel obstruction; accounting for 1%–4% of mechanical cases overall, but up to 25% in elderly patients [1]. It most frequently involves the terminal ileum, where the lumen is narrowest [2]. Colonic gallstone obstruction is exceedingly rare, with < 5% of gallstone ileus cases involving the large bowel [3]. Obstruction typically arises when a large stone passes through a bilioenteric fistula—most commonly cholecystoduodenal—and lodges at a point of narrowing, such as a diverticular stricture [4]. While surgery remains the mainstay of treatment, endoscopic techniques including mechanical lithotripsy and electrohydraulic lithotripsy (EHL) are increasingly used in patients unfit for surgery [5].

To date, only a few dozen cases of colonic gallstone ileus have been reported in the literature, with sigmoid impaction at a diverticular stricture being among the rarest presentations [3, 5]. This highlights the clinical relevance of reporting successful nonsurgical management.

## Case presentation

**Figure 1:**
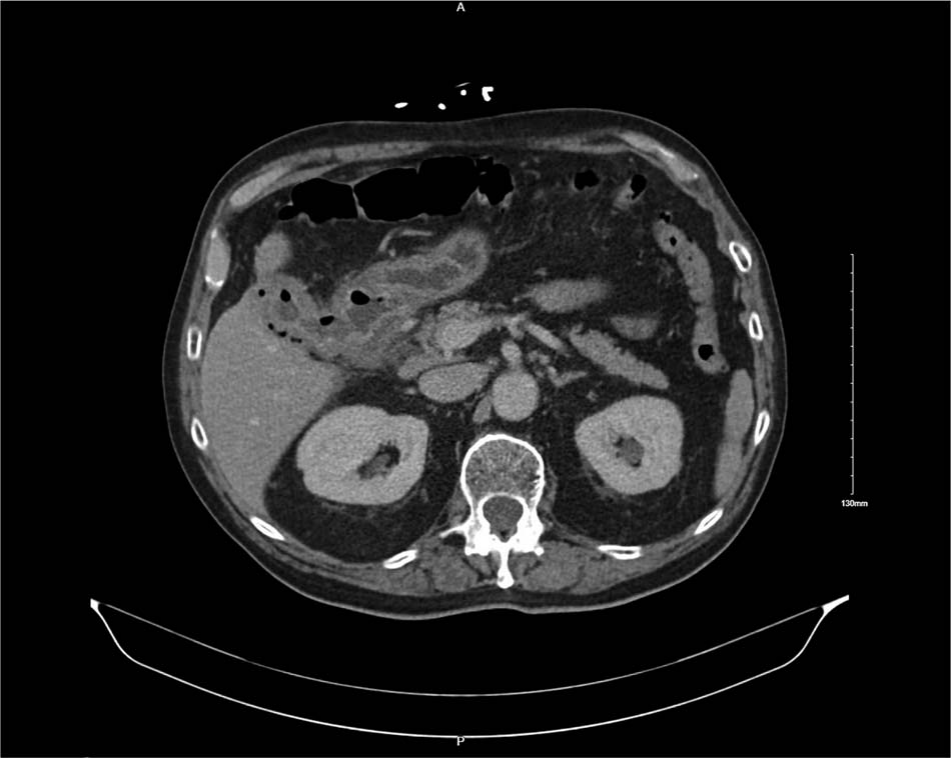
CT image showing cholocystoduodenal fistula at initial presentation.

A 76-year-old male with coronary artery disease, diastolic heart failure, atrial fibrillation on apixaban, type 2 diabetes mellitus, and chronic kidney disease presented with acute cholecystitis. Imaging revealed emphysematous cholecystitis with a suspected cholecystoduodenal fistula (Fig. 1). Cardiac workup revealed reduced EF (35%–40%) with no obstructive coronary artery disease. He was treated non-operatively with antibiotics and discharged for interval cholecystectomy.

**Figure 2:**
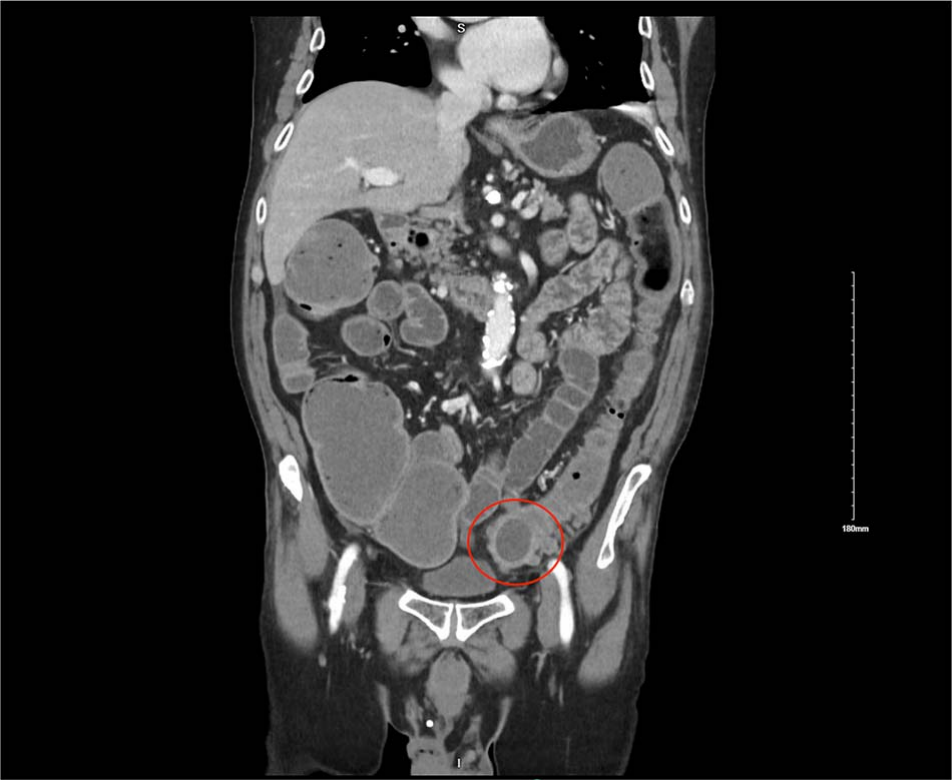
CT image showing 3 cm gallstone obstructing the sigmoid colon.

**Figure 3:**
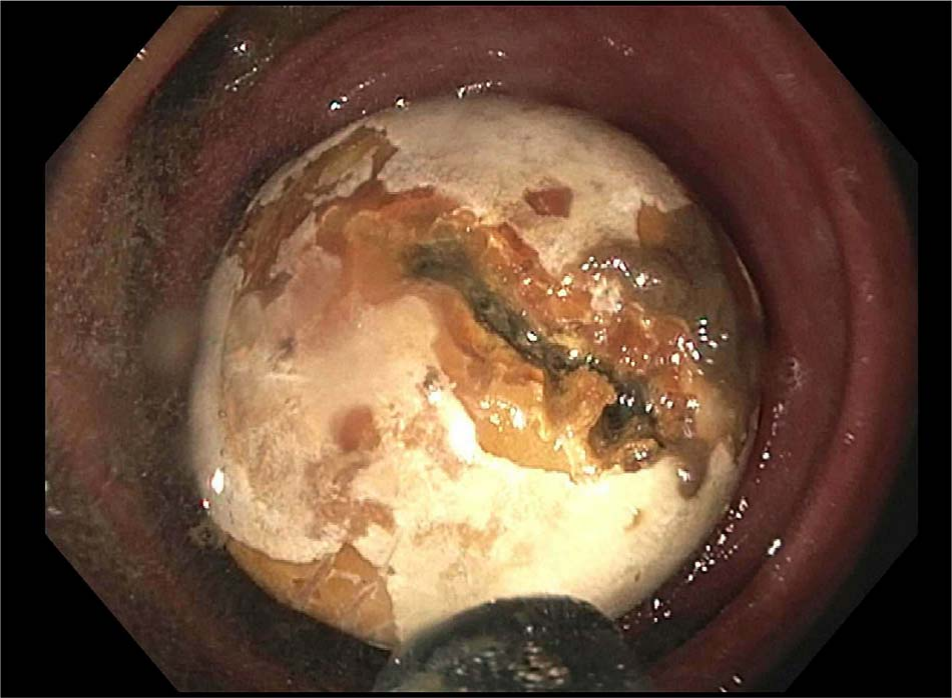
Endoscopic view of gallstone impacted at diverticular stricture.

He re-presented 10 days later with nausea, vomiting, and obstipation. CT imaging showed large bowel dilation and a 3–4 cm gallstone lodged in the proximal sigmoid colon (Fig. 2). Flexible sigmoidoscopy confirmed the presence of the stone at a diverticular stricture (Fig. 3). Initial endoscopic retrieval attempts using snare, Roth net, and EHL failed.

**Figure 4:**
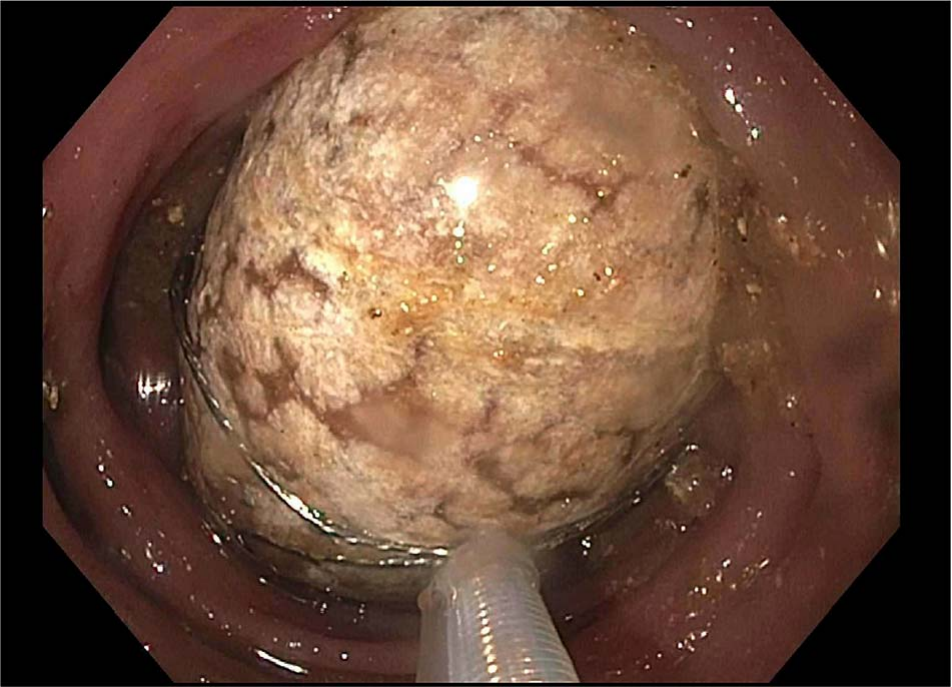
Trapezoid basket used to engage the stone.

**Figure 5:**
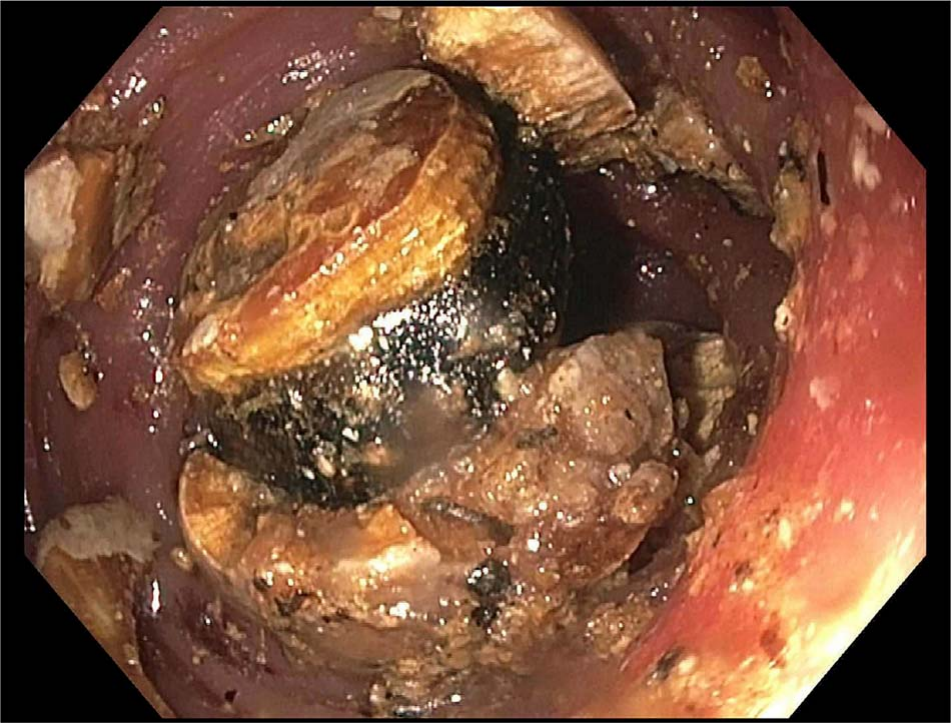
Fragmentation with mechanical lithotripsy.

**Figure 6:**
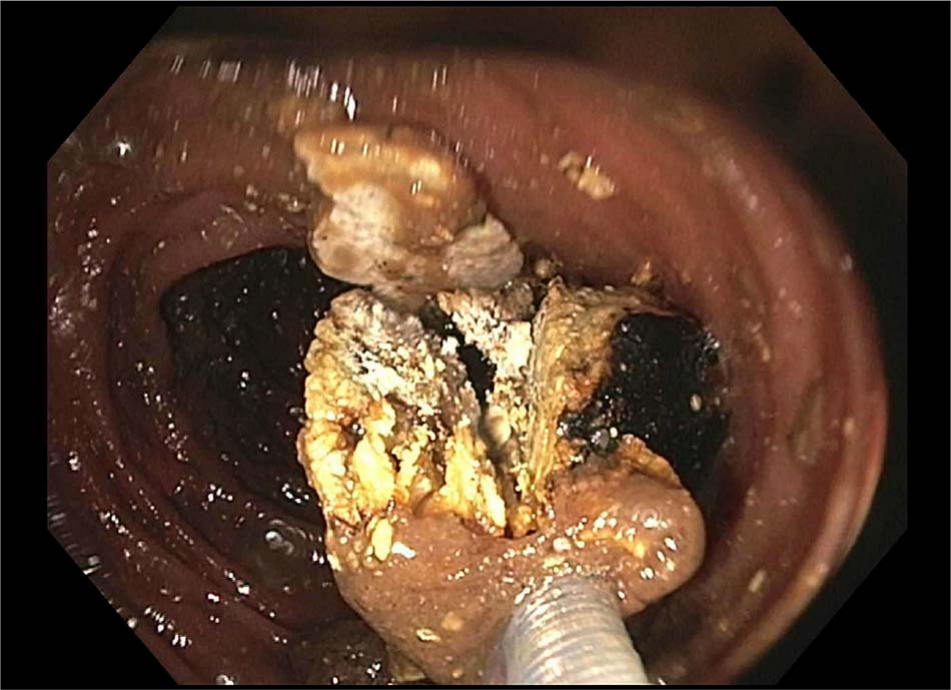
Residual gallstone with evidence of central cleavage.

A subsequent attempt with a dual-channel colonoscope allowed for mechanical lithotripsy via trapezoid basket, successfully fragmenting the stone (Figs 4–6). The patient resumed a regular diet, passed flatus and stool, and was discharged with outpatient follow-up for elective cholecystectomy.

## Discussion

Gallstone ileus remains a diagnostic and therapeutic challenge due to its rarity and the often non-specific presentation in elderly patients. While the classic radiologic Rigler’s triad—pneumobilia, ectopic gallstone, and bowel obstruction—is pathognomonic, it is not always present. The location of the gallstone determines both the clinical presentation and the treatment strategy. Involvement of the colon, especially the sigmoid segment, is particularly rare and frequently associated with underlying pathologic narrowing such as that seen in diverticulosis [3].

Traditional management has favored surgical enterolithotomy with or without cholecystectomy and fistula closure [6]. However, in high-risk patients or those with significant comorbidities, less invasive alternatives are increasingly pursued. Endoscopic management has emerged as a viable option in selected patients, particularly when the stone is accessible and the anatomy favourable [5]. In this case, the patient's cardiac status precluded major abdominal surgery, prompting an endoscopic-first approach. The use of mechanical lithotripsy allowed for fragmentation of the impacted stone, facilitating spontaneous passage and resolution of obstruction without colectomy.

This case is notable for several reasons. First, it involves a rare presentation of gallstone ileus in the colon. Second, it underscores the role of diverticular disease in predisposing to stone impaction. Finally, it demonstrates that with multidisciplinary collaboration and the use of advanced endoscopic tools, patients with significant comorbidities can be successfully managed without surgery.

## Conclusion

Gallstone-induced large bowel obstruction is a rare but serious complication that requires a high index of suspicion, particularly in elderly patients with a history of gallbladder disease. While surgery remains the mainstay of treatment for most cases of gallstone ileus, endoscopic intervention should be considered in select patients with colonic involvement and high surgical risk. This case highlights the successful use of mechanical lithotripsy to relieve an obstructing gallstone at a sigmoid diverticular stricture, thus avoiding colectomy. As endoscopic tools and techniques continue to advance, the role of minimally invasive approaches in managing rare gastrointestinal obstructions is likely to expand. Reporting such cases contributes to the growing evidence base and may support future guidelines on the management of this uncommon entity.

## References

[1] Reisner RM, Cohen JR. Gallstone ileus: a review of 1001 reported cases. Am Surg 1994;60:441–6. 8198337

[2] Inukai K . Gallstone ileus: a review. BMJ Open Gastroenterol 2019;6:e000344. 10.1136/bmjgast-2019-000344 PMC6904169 31875141

[3] Nuño-Guzmán CM, Arróniz-Jáuregui J, Méndez-Sánchez SC, et al. Gallstone ileus: one-stage surgery in a patient with intermittent obstruction. World J Gastrointest Surg 2010;2:172–6. 10.4240/wjgs.v2.i5.172 21160869 PMC2999231

[4] Halabi WJ, Kang CY, Ketana N, et al. Surgery for gallstone ileus: a nationwide comparison of trends and outcomes. Ann Surg 2014;259:329–35. 10.1097/SLA.0b013e31827eefed 23295322

[5] Dumonceau JM, Devière J. Novel treatment options for Bouveret’s syndrome: a comprehensive review of 61 cases of successful endoscopic treatment. Expert Rev Gastroenterol Hepatol 2016;10:1245–55. 10.1080/17474124.2016.1241142 27677937

[6] Clavien PA, Richon J, Burgan S, et al. Gallstone ileus. Br J Surg 1990;77:737–42. 10.1002/bjs.1800770707 2200556

